# Substituent Effects on EI-MS Fragmentation Patterns of 5-Allyloxy-1-aryl-tetrazoles and 4-Allyl-1-aryl-tetrazole-5-ones; Correlation with UV-Induced Fragmentation Channels

**DOI:** 10.3390/molecules26113282

**Published:** 2021-05-29

**Authors:** Alina Secrieru, Rabah Oumeddour, Maria L. S. Cristiano

**Affiliations:** 1 CCMAR and Department of Chemistry and Pharmacy, FCT, Campus de Gambelas, University of Algarve, 8005-039 Faro, Portugal; asecrieru@ualg.pt (A.S.); oumeddour.rabah@univ-guelma.dz (R.O.); 2 Department of Chemistry, University of Liverpool, Liverpool L69 7ZD, UK; 3 Laboratory of Industrial Analysis and Materials Science, Faculty MISM, University 8 Mai 1945, Guelma 24000, Algeria

**Keywords:** 5-allyloxy-1-aryl-tetrazoles, 4-allyl-1-aryl-tetrazole-5-ones, mass spectrometry, electron impact, fragmentation patterns, matrix isolation, UV-induced fragmentation, substituent effects

## Abstract

1,4- and 1,5-disubstituted tetrazoles possess enriched structures and versatile chemistry, representing a challenge for chemists. In the present work, we unravel the fragmentation patterns of a chemically diverse range of 5-allyloxy-1-aryl-tetrazoles and 4-allyl-1-aryl-tetrazolole-5-ones when subjected to electron impact mass spectrometry (EI-MS) and investigate the correlation with the UV-induced fragmentation channels of the matrix-isolated tetrazole derivatives. Our results indicate that the fragmentation pathways of the selected tetrazoles in EI-MS are highly influenced by the electronic effects induced by substitution. Multiple pathways can be envisaged to explain the mechanisms of fragmentation, frequently awarding common final species, namely arylisocyanate, arylazide, arylnitrene, isocyanic acid and hydrogen azide radical cations, as well as allyl/aryl cations. The identified fragments are consistent with those found in previous investigations concerning the photochemical stability of the same class of molecules. This parallelism showcases a similarity in the behaviour of tetrazoles under EI-MS and UV-irradiation in the inert environment of cryogenic matrices of noble gases, providing efficient tools for reactivity predictions, whether for analytical ends or more in-depth studies. Theoretical calculations provide complementary information to articulate predictions of resulting products.

## 1. Introduction

Tetrazole [1] and derivatives have attracted perennial attention for several decades due to their structural features and unique reactivity traits, as well as relevant applications [2,3,4,5,6,7]. Among the main features responsible for the wide array of applications of tetrazoles are the acid/base and electron-withdrawing properties of the heteroaromatic ring. In tetrazole and 5-substituted tetrazoles, the presence of three tertiary nitrogen atoms together with a free NH group in the heterocyclic moiety leads to an acidic character comparable to that of aliphatic carboxylic acids [8]. It has been held that 5-substituted-1*H*-tetrazoles (RCN_4_H) may serve as nonclassical isosteres for the carboxylic acid moiety (RCO_2_H) in biologically active molecules, providing an additional advantage in metabolic stability at physiologic pH [9,10,11,12]. In addition, the electron-withdrawing properties of tetrazole have been put to use in the development of synthetic methodologies for the reductive cleavage of the C-OH bond in alcohols [13,14,15].

From a different perspective, the acid/base properties of tetrazoles also raise the possibility of intramolecular rearrangements, namely prototropic tautomerism, which may influence the reactivity of the class in regard to electronically induced cleavages, as observed photochemically [16,17,18]. 1,5- or 2,5-disubstituted tetrazoles circumvent the possibility of tautomerism, but the reactivity then remains highly dependent on the nature of substituents [12,19,20].

Mass spectrometry is an analytical technique based on the ionization and fragmentation of molecules as an instrument to determine masses and elucidate their structure. Electronic ionization (or electron impact, EI) relies on the incidence of electrons as the energy source to promote transformations in the analytes, whether these are cleavages or rearrangements. Analogously to EI-MS fragmentations, photochemical transformations rely on the light-promoted excitation of molecules. Isolation of the analytes in cryogenic noble gas matrices confers the possibility to create an inert environment, impeding the occurrence of cross-reactions. Chemical modifications in the compounds subjected to the experiment can then be followed by an adequate probing method, such as Infrared (FTIR) spectroscopy, thereby enabling the elucidation of fragmentation channels and characterization of photoproducts [21].

While the photochemistry of tetrazoles has been the subject of several investigations, EI-MS studies on fragmentation patterns of tetrazoles are very scarce. In particular, the fragmentation patterns of 5-allyloxy-1-aryl-tetrazoles and 4-allyl-1-aryl-tetrazolole-5-ones when subjected to electron impact mass spectrometry (EI-MS) have not been thoroughly explored. In a study of the fragmentation patterns of 1,5-disubstituted tetrazoles, Fraser et al. showed that tetrazoles carrying methyl and aryl substituents at positions 1 and 5, respectively, fragment essentially in two different manners, namely through the extrusion of CHN_2_ or N_3_ and through the elimination of N_2_ or CHN [22]. N_2_ elimination was also shown to be the preferential primary fragmentation process during studies conducted by Fischer et al. and Shurukhin et al., on 5-(1-acyl-2-dialkylaminovinyl)-1-aryl-1H-tetrazoles and 1-(*o*-, *m*-, and *p*-tolyl)-5-phenyltetrazoles, respectively [23,24]. Nevertheless, although some important features are frequently retained, one important conclusion to withhold is that electron ionization-induced cleavages in this class of compounds are highly dependent upon the nature of the substituents [22,23,25,26]. In parallel, studies on the photochemistry of some substituted tetrazoles have shown that the preferential structures formed from photocleavage are azides, aziridines and isocyanates, with the nature of the substituents attached to the tetrazole ring strongly influencing the fate of photoreactions, whether due to their chemical nature, the possibility of cross-reactions or conformational impairments [16,27,28,29].

In an attempt to further explore the influence of substituents on the excited state reactivity of some disubstituted tetrazoles, we performed an EI-MS study on a library of 5-allyloxy-1-aryl-tetrazoles and their products of Claisen-type isomerization, 4-allyl-1-aryl-tetrazole-5-ones. The results gathered from mass spectra analysis provide accurate identification of the species formed upon labile bond cleavage of the studied tetrazoles when ionised through electronic impact, as well as the assessment of divergent cleavage patterns in the function of the diversified nature of the substituents. Furthermore, the results gathered using the EI-MS technique are compared with those obtained from UV-induced photolysis of monomeric tetrazoles isolated in cryogenic matrices of noble gases [12,30] to further assess the degree of comparability between the fragmentation patterns obtained using the two forms of excitation, as this analysis can provide a useful complementary technique in reactivity studies of this class of molecules and possibly others.

## 2. Results

**Scheme 1:**
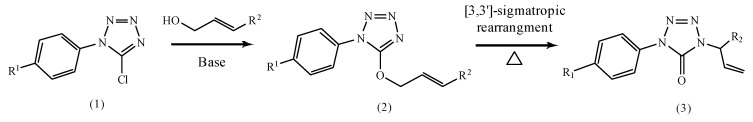
A schematic representation of the synthetic route to 5-allyloxy-1-aryl-tetrazoles (**2**) and 4-allyl-1-aryl-tetrazole-5-ones (**3**), from 5-chloro-1-aryl-tetrazoles (**1**).

A library of 5-allyloxy-1-aryl-tetrazoles 2a–h and 4-allyl-1-aryl-tetrazole-5-ones 3b-h was studied to elucidate the behaviour of the compounds when subjected to electronically induced fragmentation in EI-MS. 5-allyloxy-1-aryl-tetrazoles 2 were prepared from the reaction of 5-chloro-1-aryl-tetrazoles 1 with the respective allyl alcohol in an alkaline medium, as depicted in Scheme 1. When heated neat, at 100 °C, allyl tetrazolyl ethers 2 rearrange to give exclusively the corresponding tetrazolones 3, the process occurring through a concerted [3,3′]-sigmatropic isomerisation (Scheme 1) [31,32]. Hence, 4-allyl-1-aryl-tetrazole-5-ones 3 are prepared by thermal rearrangement of their 5-allyloxy-1-aryl-tetrazole precursors 2.

Within each set of tetrazoles studied, allyl tetrazolyl ethers and corresponding tetrazolones, two main subsets were explored (Table 1): in one, the substituent at R^1^ was maintained, and the substituents at R^2^ were varied; in the other, the opposite effect was evaluated, i.e., the substituent at R^2^ was kept constant while varying the substituents at R^1^. The results are presented in Table 1 and Table 2, and the corresponding spectra are provided as supplementary data.

### 2.1. 5-Allyloxy-1-aryl-tetrazoles

Initially, we analysed the allyl tetrazolyl ether series, i.e., 5-allyloxy-1-aryl-tetrazoles 2a–h (Table 1). In this group, the first subset of compounds, 2a–e, is characterised by the presence of a phenyl group at position 1 of the tetrazole ring (R^1^ = H), and variable R^2^ at the allylic side-chain (R^2^ = H (2a), CH_3_ (2b), C_6_H_5_ (2c), C_7_H_12_ (2d) and C_6_H_4_NO_2_ (2e)), whereas the second subset of the series, 2f-h, is composed of a constant R^2^ group (R^2^ = C_6_H_5_) and different substituents at the position 4 of the N1 phenyl group (R^1^ = NO_2_ (2f), F (2g) and NH_2_ (2h)). Table 1 gathers detailed information about the structure of 5-allyloxy-1-aryl-tetrazoles 2a–h and the fragments produced by each molecule. For the sake of consistency and precision, peaks were solely considered significant at intensities equal or superior to 5% relative to the base peak, and only the relevant peaks to this class of compounds were shown. The peaks with *m*/*z* values attributed to the cleavage of phenyl and other well-known structures were omitted.

### 2.2. 4-Allyl-1-aryl-tetrazole-5-ones

In continuation of the study, we evaluated the behaviour of the series composed of the 4-allyl-1-aryl-tetrazole-5-ones 3b-h. This series of tetrazoles, similarly to the former, was divided into two subsets: one in which the phenyl group at N1 remained unsubstituted, R^1^ = H, and the substituents at R^2^ varied (R^2^ = CH_3_ (3b) and C_6_H_5_ (3c)), and a second one in which R^2^ was kept constant (R^2^ = C_6_H_5_), differing the substituents at R^1^ (R^1^ = NO_2_ (3f), F (3g) and NH_2_ (3h)). Table 2 summarises the information for the 4-allyl-1-aryl-tetrazole-5-one series under study and the respective spectral results.

## 3. Discussion

### 3.1. Electron Impact Mass Spectrometry

#### 3.1.1. Fragmentation Patterns of 5-Allyloxy-1-aryl-tetrazoles

**Scheme 2:**
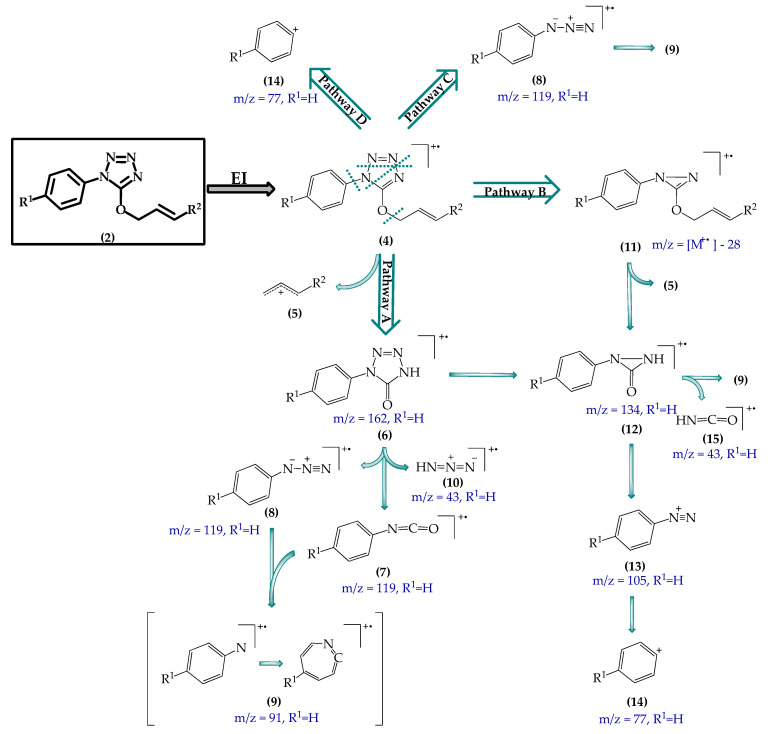
A schematic representation of the proposed cleavage channels for the studied library of 5-allyloxy-1-aryl-tetrazoles. The black arrow depicts the electron impact process. Green arrows represent cleavage steps. Blue letters refer to the masses of each fragment when unsubstituted at R^1^ (R^1^ = H).

Upon bombardment of each tetrazole molecule with high-energy electrons, an electron is lost from one of the HOMO orbitals, originating the molecular ion, [M^+·^] (structure 4, Scheme 2). With sufficiently high applied energies, the intensities of the molecular ion peaks tend to drop due to multiple fragmentations, while a diverse set of smaller fragments appears in the medium. The low-energy bonds are usually the most affected by fragmentation, and the most intense peaks correspond to the most stable fragments.

In 5-allyloxy-1-aryl-tetrazoles, the specific site of initial ionization cannot be precisely determined because this chemotype possesses several groups with similar ionization energies, making it difficult to ascertain the exact orbital from which the initial electron should be ejected. Additionally, the non-bonding electrons of the heteroatoms in these molecules contribute to the possibility of resonance stabilisation in the molecular ion, delocalising the radical cation throughout the tetrazole core and the adjacent oxygen atom. These features enable the molecular ion to undergo multiple fragmentation pathways, determined by the positions carrying the unfilled orbitals and the stability of the resulting even or odd-electron ion and respective radical or neutral species. As such, from the evaluation of the EI-MS spectra, we propose the primary fragmentation processes of the 5-allyloxy-1-aryl-tetrazoles molecular radical cation 4 (Scheme 2) to proceed via four main pathways, hereinafter referred to as pathways A, B, C and D, as depicted in Scheme 2. The highly electronegative nature of the tetrazole ring weakens the C–O bond connecting this ring to the allylic fragment, hence promoting an immediate loss of the allylic fragment. In pathway A, it can lead to an allylic carbocation specific to each substituent at R^2^ (structure (5), Scheme 2) and to a 1-aryltetrazolone radical cation (structure (6), Scheme 2). The latter can then undergo further cleavage, leading to several important secondary structures: the loss of N_2_ generates a diaziridinone intermediate (structure (12), Scheme 2), providing a convergence step with another cleavage pathway, pathway B, which results from direct N_2_ loss as a primary fragmentation step into a diazirine radical cation (structure (11), Scheme 2), prior to the loss of the allylic fragment yielding structure (12). The convergence of routes A and B leads to the formation of three important fragments frequently identified in the mass spectra of this class, a *p*-aryldiazene cation (structure (13), Scheme 2), an aryl cation (structure (14), Scheme 2) and an isocyanic acid radical cation (structure (15), Scheme 2).

The other secondary routes from pathway A result from the opening of the tetrazole cycle. This process entails the development of two main subproducts with the same mass, an aryl azide (structure (8), Scheme 2) and an aryl isocyanate (structure (7), Scheme 2). Further loss of neutral small molecules N_2_ and CO from the azide and isocyanate derivatives, respectively, results in the generation of an aryl nitrene structure (structure (9), Scheme 2). In mass spectrometry, cyclic aromatic species carrying a single charged group attached to them, such as toluene and other benzyl analogues, frequently undergo a ring expansion process leading to more charge-stabilising seven-membered ring systems [33]. Previous EI-MS works on phenyl azide and analogues [34], including recent computational studies on tetrazole derivatives, at DFT and CASPT2 (7,9) levels of theory [35,36], aimed at determining if the same could be observed for aryl nitrenes, corroborated this proposal for phenyl nitrene (*m*/*z* 91) and other substituted analogues. Hence, it appears reasonable to consider that the same process takes place for the product at *m*/*z* 91 and its substituted analogues, the phenyl nitrene radical cation being subjected to a ring expansion process to produce a more stable chemical entity, a 1-aza-1,2,4,6-cycloheptatetraene (ACHT) derivative.

Apart from the aforementioned pathways, we propose the presence of two additional primary cleavage channels for 5-allyloxy-1-aryl-tetrazoles, pathways C and D, leading directly to some of the fragments formed in pathways A and B. Pathway C should then promote the formation of the aryl azide compound (**8**) as a primary fragment, further breaking into (9) and CO (Scheme 2). Pathway D, however, represents the immediate detachment of the aryl substituent at N1 from the tetrazole core, allowing for the phenyl derivative to arise as a product of a primary fragmentation process.

In a different light, although the cleavage pathways A-D are common to 5-allyloxytetrazoles 2a–h, from the analysis of the spectra of each individual tetrazole of the class, distinct pathways seem to characterize the primary routes of fragmentation, based on the substitution patterns at R^1^ and R^2^. In compounds **2**a–**2**e R^1^ = H, any variations in fragmentation patterns result from the nature of the R^2^ substituent, whether due to electronic or steric effects. Thus, it is worthwhile exploring the variabilities within this group firstly.

In compounds **2**a and **2**b, the structures vary solely by the addition of a methyl group to the allylic substituent in 2b. The behaviour of such similar compounds should then be expected to be similar; however, the results seem to indicate otherwise. In compound **2**a, the base peak is seen at *m*/*z* 77, which indicates the phenyl cation as the most abundant fragment. The following peak, with *m*/*z* 41 and 92% intensity, corresponds to the propene cation, which can form upon cleavage of the C–O bond as a primary fragmentation step in pathway A, or following the loss of a nitrogen molecule from the tetrazole core, in pathway B. The fragments with *m*/*z* 119 and *m*/*z* 91 are also formed in high proportions, indicating cleavage of the tetrazole core to give a phenyl azide and/or phenyl isocyanate species, as well as the phenyl nitrene structure that succeeds the cleavage of both species, but these are common to more than one pathway and hence it is important to seek additional guides towards the main fragmentation channels of this compound. The spectrum shows two important peaks for this determination, the peaks at *m*/*z* 173 and 133. These peaks appear to indicate the loss of N_2_ from the molecular ion through pathway B (structure (11), Scheme 2), followed by the loss of the allylic fragment (structure (12), Scheme 2), respectively. Interestingly, a hydrogen atom leaves the molecule at the initial fragmentation step, in keeping with previous observations during the analysis of the mass spectra of other 1,5-disubstituted tetrazoles [22]. Since pathway B culminates in the formation of phenyl and includes the loss of the propene cation, this pathway seems to be the preferred one for cleavage in 2a. The absence of a fragment at *m*/*z* 162 (accounting for [M^+·^]-41 in pathway A) appears to corroborate this theory, suggesting that the fragments at *m*/*z* 119 and 91 should arise from direct breakage of the tetrazole core through pathway C. Therefore, we propose that the main fragmentation route for this compound is pathway B, followed by pathway C.

Compound **2**b bears a methyl group at R^2^. The spectrum of 2b shows a base peak at *m*/*z* 119, a fragment at *m*/*z* 55 with 60% abundance and less abundant fragments at *m*/*z* 77, 91 and 162. In this case, the main species resulting from ionisation of 2b correspond to the structures (7) and/or (8) (Scheme 2), which can arise from cleavage through pathways A or C. However, the spectrum shows a small (yet significant) peak at *m*/*z* 162, ascribed to 1-phenyl-5-tetrazolone ([M^+·^]-55; structure (6), Scheme 2), supporting pathway A as the preferred primary fragmentation process for 2b. Compared to compound **2**a, the methyl group in 2b emerges as the stabilising factor for the allylic cation and neutral leaving group, also favouring the cleavage at the ether moiety through pathway A.

In compound **2**c, a similar behaviour to 2b is observed regarding a stabilisation by the R^2^ group, the conjugated π system of the phenyl providing an even stronger stabilisation of the allyl cation through resonance. The spectral data for derivative 2c shows the fragment representing the allylic segment appearing in the medium as a base peak at *m*/*z* 117 and very low abundancies of any of the other fragments, indicating α-cleavage at the ether function towards the formation of the allylic cation (structure (5), Scheme 2) through pathways A and/or B. In this case, for the sake of pathway elucidation, a peak at *m*/*z* 250 with 4% intensity was also considered relevant. This peak represents the segment [M^+·^]-28 (structure (11), Scheme 2), indicating extrusion of a nitrogen molecule from the tetrazole core. As such, a contribution from cleavage through pathway B, with the elimination of N_2_ prior to the α-cleavage at the ether bond, should not be discarded.

Compound 2d bears a bulkier substituent at R^2^, but in this case, lacking the additional stabilisation from the aromatic π system. From the spectral data, compound **2**d demonstrates a higher degree of fragmentation comparatively to the aforementioned counterparts, with higher abundances of fragments from all four pathways. The base peak at *m*/*z* 119, followed by the *m*/*z* 91 peak at 80%, suggests pathways A or C as primary fragmentation routes; the presence of 56% abundance of the diaziridinone fragment at *m*/*z* 134 (structure (12), Scheme 2) indicates the possible contribution of pathway B through immediate loss of N_2_; the allylic fragment at *m*/*z* 163 (structure (5), Scheme 2) with 43% indicates high stability of the allylic fragment and cleavage through pathway A or B toward the formation of this cation; the peak at *m*/*z* 77 representing the phenyl group (structure (14), Scheme 2) with 40% abundance reinforces the possibility of various cleavage mechanisms since this fragment can arise from pathways A and B and also from direct cleavage through pathway D. Therefore, from the gathered data, compound **2**d emerges as a more versatile tetrazole derivative with respect to the fragmentation patterns upon electronic ionization, yielding a variety of abundant fragments.

Compound **2**e is structurally identical to 2c, with the addition of a nitro electron-withdrawing group (EWG) at position 4 of the phenyl at R^2^. Interestingly, the major peaks in the spectrum of 2e are also the ones present in 2c; however, the ratios of fragments differ, reflecting the implication of the nitro group in the fragmentation routes followed by compound **2**e. While compounds **2**a–d carry electron-donating groups at R^2^ (whether through resonance or inductive effects), acting as stabilising agents, the presence of an EWG at the R^2^ phenyl in compound **2**e is expected to contribute to the weakening of the C–O bond, inducing cleavage at the indicated position. Contrariwise, the presence of the NO_2_ group in the allylic group is expected to disrupt the charge stabilisation in the cationic allyl fragment. Pathway A should be preferred, with the formation of the aryl-tetrazolone radical cation (structure (6), Scheme 2) as a favoured fragment. The base peak in the spectrum of this compound appears at *m*/*z* 119, followed by the peaks at *m*/*z* 116, 91, 77 and 43, corresponding to structures (6) and/or (7), (4), (8), (13) and (9) and/or (14) of Scheme 2, respectively. The peak at *m*/*z* 116 indicates the loss of the NO_2_ group from the allylic substituent, providing higher stability of the final structures. Thus, we propose that pathway A is the primary fragmentation process upon electronic ionization for compound **2**e.

Compounds **2**f–h are structurally different, as, in this subset of 5-allyloxy-1-aryl-tetrazoles, the structural features at R^2^ are maintained, varying the nature of the substituent at R^1^. When comparing ethers 2f–h, the electronic nature of the substituents stands out: compounds **2**f and **2**g carry nitro and fluoro substituents, respectively, hence exerting an electron-withdrawing effect on the phenyl group at N1, while 2h carries an amino group, acting as an electron donor to the phenyl, these effects expanding to the tetrazole ring.

The EI-MS spectrum of 5-(cinnamyloxy)-1-(4-nitrophenyl)-1*H*-tetrazole, 2f, only shows five significant peaks, at *m*/*z* 117, 118, 91, 295 and 77. The peak with *m*/*z* 117 represents the base peak, with 100% abundance, indicating that the stable cinnamyl cation favours cleavage at the ether moiety, this being the main cleavage site in this compound. As described previously, this cleavage occurs in pathways A and B, and it is therefore important to determine the primary process leading to the cinnamyl fragment. The spectrum suggests initial loss of N_2_ with the formation of species (11) (Scheme 2), evidenced by the presence of a peak at *m*/*z* 295, which indicates that pathway B is the primary route in this case. The peak at *m*/*z* 77 reinforces this hypothesis, since this fragment also takes part in this cleavage process. Besides pathway B, the data shows a contribution of at least one additional pathway, as the fragment with *m*/*z* 118 is believed to belong to the phenyl azide formed in pathway C subsequent to the loss of the nitro group. This fragment should then cleave to form the phenyl nitrene/1-aza-1,2,4,6-cycloheptatetraene pair, with *m*/*z* 91. Nonetheless, despite the presence of small amounts of the aforementioned products, the cinnamyl moiety as the most abundant species is an indicator that the stronger electron-withdrawing force imposed by the nitro group to the already electronegative tetrazole core contributes to the elongation of the labile C–O bond so that its cleavage with the formation of the stabilised cinnamyl cation prevails overwhelmingly.

Similar results are observed in the spectrum of 2g, 5-(cinnamyloxy)-1-(4-fluorophenyl)-1*H*-tetrazole, which also shows the base peak at *m*/*z* 117 and a small peak showing the loss of the N_2_ molecule, indicating a preference for fragmentation through pathway B with the formation of the cinnamyl fragment. In this case, however, the weaker electron-withdrawing nature of the fluorine atom comparatively to the nitro group enables a more versatile fragmentation of the molecule, leading to other abundant species in the medium. Fragments with *m*/*z* at 137 and 109 with significant abundances show cleavage of the tetrazole core also through pathway C, these fragments corresponding to the phenyl azide species and the phenyl nitrene/1-aza-1,2,4,6-cycloheptatetraene pair, respectively, also observed for compound **2**g. The subsequent loss of the fluorine affords fragments with *m*/*z* 118 and 91, respectively, and some residual amount of the phenyl cation, with *m*/*z* 77.

1-(4-aminophenyl)-5-(cinnamyloxy)-1*H*-tetrazole, 2h, carrying the electron-donating NH_2_ group at N1-phenyl, behaves differently from the EWG-substituted counterparts. The base peak in this compound is observed at *m*/*z* 134, raising several possibilities for fragmentation pathways. One hypothesis would be that the mesomeric stabilisation provided by the amino group causes the group to remain attached to the tetrazolic phenyl and steer the cleavages towards the other labile bonds in the structure. This way, the fragment at *m*/*z* 134 should correspond to the 4-aminophenyl azide radical cation yielded by the direct split of the tetrazole ring through pathway C, which can subsequently cleave into a 4-aminophenyl nitrene at *m*/*z* 106 through the loss of a neutral nitrogen molecule, giving rise to the second-highest peak in the spectrum. From a different perspective, however, an interesting feature is observed in the spectrum of this compound. A small peak with *m*/*z* 250, representing [M^+·^]-43, seems to indicate the loss of HN_3_ from the tetrazole core, a feature frequently observed in tetrazoles, which, in the case of our library of compounds, usually occurs only after the previous α-cleavage at the ether function. The presence of this peak can indicate that the fragment at *m*/*z* 134 might belong to the 4-aminophenyl isocyanate structure that results from the elimination of the cinnamyl group from the species at *m*/*z* 250. From yet another point of view, another species could be attributable to the compound with *m*/*z* 134, which is the diaziridinone radical cation 12 formed through B. However, the spectral data shows no trace of the three-membered precursor diazirine cycle represented as structure (11) in Scheme 2, indicating that a loss of N_2_ might not occur for this compound, contrary to the previous two compounds carrying electron-withdrawing groups at position 4 of the N1 phenyl substituent.

#### 3.1.2. Fragmentation Patterns of 4-Allyl-1-aryltetrazole-5-ones

**Scheme 3:**
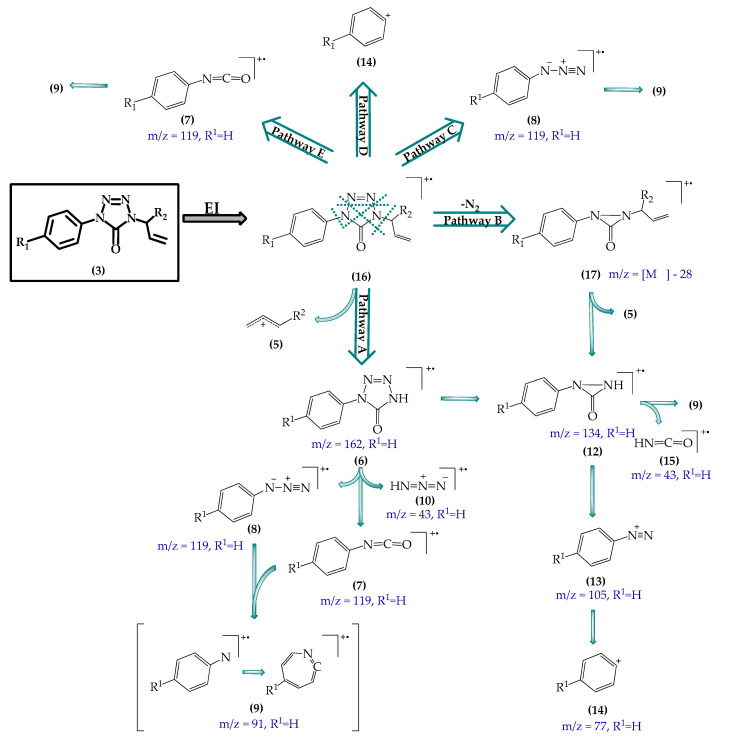
A schematic representation of the proposed cleavage channels for the studied library of 4-allyl-1-aryltetrazole-5-one derivatives. The black arrow depicts the electron impact process. Green arrows represent cleavage steps. Blue letters refer to the masses of each fragment when unsubstituted at R^1^ (R^1^ = H).

To shed light on the fragmentation processes of the tetrazolone series, the EI-MS spectra of this group were evaluated. It is known that 5-allyloxy-1-aryltetrazoles are characterised by the presence of a labile C–O bond, which is the bond that cleaves upon heating, leading to the respective tetrazolones (Scheme 1). As the labile ether function is no longer present in tetrazolones, the pathway leading to allylic fragments would imply cleavage of the C–N bond linking the allyl moiety and the tetrazole ring. However, an important feature to consider is that, in our study, the tetrazolone analogues were obtained by thermal sigmatropic rearrangement of the respective tetrazolyl ethers. Taking into consideration that the EI-MS technique involves a heating step as an initial operation for electronic ionization, one must not overlook the possibility of an initial isomerisation of the ethers to tetrazolones and the possibility of identical results detected in both series of tetrazoles under investigation. As observed for the previous group, upon high-energy electron impact, the ionization process starts by the ejection of one electron from the original molecule to form a molecular radical cation (structure (16), Scheme 3). Regarding the fragmentation pathways of 4-allyl-1-aryltetrazole-5-ones, the results collected from the spectral data led us to propose five pathways of primary fragmentations, referred to as pathway A, B, C, D and E. Interestingly, our main observation from the analysis of the results was that the structural similarities between the tetrazolyl ethers and their ketone analogues appear to promote similar types of fragmentation in both groups, as well as the formation of many common fragments, and the main difference lies in the possibility of immediate breakage of the tetrazole core through pathway E, leading to the phenyl isocyanate fragment (7) (Scheme 2 and Scheme 3). Unlike for the previous group, however, the aromatic nature of the tetrazole ring is lost, the labile C–O bond is also lost, and a stronger C–N bond is formed. Therefore, it is interesting to explore whether these features play a significant role in the breakage patterns of this class compared to the previous. Scheme 3 depicts the proposed fragmentation channels for the studied 4-allyl-1-aryltetrazole-5-ones.

Hereafter, the detailed description of each tetrazolone of this set is presented. Tettrazolone 3b, 4-(but-3-en-2-yl)-1-phenyl-1H-tetrazol-5(4H)-one, represents the functional isomer of ether 2b. The mass spectrum of 3b shows four important peaks, the base peak at 119, followed by the peaks at *m*/*z* 91, 55 and 77, with intensities of 23, 14 and 11, respectively. These results indicate that compound **3**b cleaves yielding the fragment with *m*/*z* 119 almost exclusively, which, as said above, can correspond to a phenyl azide (structure (8), Scheme 2 and Scheme 3) or to a phenyl isocyanate (structure (7), Scheme 2 and Scheme 3). Formation of these compounds could ensue from the generic pathways A, C and E, but, in this particular case, pathway A should not contribute to the primary fragmentation processes, given the absence of any traces of the peak at 162 accounting for the loss of the allylic substituent, [M^+·^]-55 (observed for 2b). This could be explained by the strength of the C–N bond as well as the higher fragility of the tetrazole core. Therefore, pathways C and E should be the main fragmentation routes for tetrazolone 3b.

Tetrazolone 3c bears a cinnamyl substituent at R^2^. The spectral behaviour of this compound is identical to that of its ether analogue 2c. The base peak shows at *m*/*z* 117, and the percentages of the other fragments are almost negligible, suggesting that the major breakage occurs at the C–N bond from position 4 of the tetrazole core. Although it should be expected that the stronger C–N bond in 3c alters the fragmentation behaviour comparatively to 2c, the highly stabilised nature of the cinnamyl group seems to be the driving force for its formation. Additionally, as seen for 2c, in this case, the fragment at 250 is also present, indicating that pathway B plays an important role in the fragmentation of 3c.

The remaining tetrazolones analysed, 3f, 3g and 3h, correspond to the study group in which R^1^ differs. Tetrazolone 3f carries a 4-nitrophenyl group at N1. The EI mass spectrum of 3f shows four main peaks, from which the fragment with *m*/*z* 117 represents the base peak, with 100% abundance. This fragment corresponds to the allylic cinnamyl group (structure (5), Scheme 2 and Scheme 3), arising from cleavage of the bond between the N4 atom of the tetrazole core and the adjacent carbon of the allylic substituent. The fragment at 118 seems to correspond to structures (7) and/or (8) after the loss of NO_2_, indicating a tendency towards the genesis of the more stable unsubstituted forms of the phenyl azide and isocyanate structures. Nevertheless, despite the higher stability, low concentrations of these are detected in the mass spectrum of this compound, suggesting that the main position for cleavage is the C–N bond at the tetrazole N4.

Compound **3**g bears fluorine as a substituent at R^1^, which is also an EWG, as in tetrazolone 3f. Thus, the fragmentation pattern of 3g is practically identical to 3f, the main difference being the relative percentage of the aryl azide and/or isocyanate entities, still carrying the substituent at R^1^, yielding a peak at *m*/*z* 137. This difference is probably due to the comparatively weaker electron-withdrawing capacity of the substituent.

Lastly, compound **3**h carries the electron-donating amine (NH_2_) at position 4 of the N1 phenyl group. As for the 2h analogue from the previous group, the pattern of fragmentation alters significantly in comparison to the previous two compounds. Five significant peaks are observed in the spectrum of 3h (*m*/*z* 134, 117, 106, 91 and 43). From the interpretation of the spectrum of 2h, it is possible to observe that, besides small variations in the percentages of the less abundant fragments in the spectrum, the molecules behave almost identically. In 3h, however, the percentage of the compound with *m*/*z* 106 decreases significantly compared to 2h (indicating that an allyl ether structure might play a role); thus, pathway E could be the preferred route for this compound, given the presence of a base peak at *m*/*z* 134 corresponding to the 4-aminophenyl isocyanate.

### 3.2. Photochemical Studies of Matrix-Isolated Tetrazoles

Prior to the EI-MS evaluation of our library of tetrazoles, photochemical studies of selected tetrazoles in matrix isolated conditions were performed, and the light-induced fragmentation patterns were scrutinized. The results from the photochemical studies assisted us in elucidating the structures of fragments obtained upon electronic impact.

Matrix isolation is a technique that can be utilised for studying the structure and reactivity of molecules in an inert environment, usually solidified noble gases at low temperatures, where cross-reactions and intramolecular rearrangements are prevented by the rigid and inactive nature of the matrix. When coupled to a suitably analytical technique, usually FTIR spectroscopy, the method allows for an accurate study of reactive and/or unusual species which in other environments are labile or prone to prompt modifications, impeding their spectroscopic or spectrometric detection [37]. This feature allows for more accurate identification of the species, also facilitating the correlation between the products detected from each of the analytical techniques used.

The results gathered in our studies show that a correlation can be established between the fragmentation patterns of our series of tetrazoles in mass spectrometry under electron impact and those achieved upon exposure to UV radiation. Identification of fragments obtained by EI-MS indicates that the fragmentation channels coincide with those identified in previous studies undertaken on the matrix-photochemistry of the same molecules or molecules from the same class, which we present in detail hereinafter.

#### 3.2.1. Tetrazolyl Ethers

**Scheme 4:**
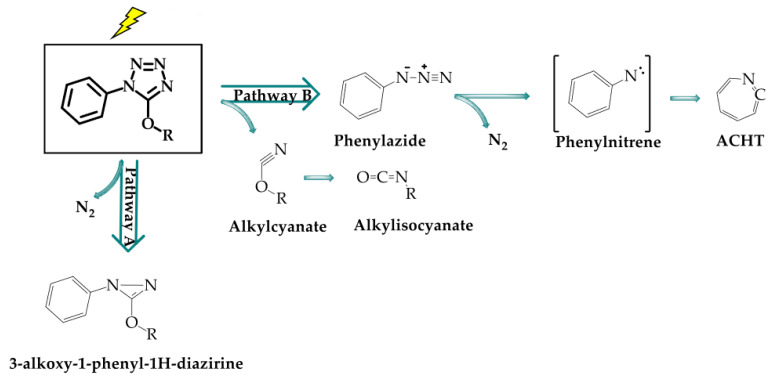
A schematic representation of the proposed fragmentation pathways for matrix isolated 5-alkoxy-1-phenyl-1H-tetrazoles irradiated with UV light at *λ* > 235 nm.

Three tetrazolyl ethers were subjected to light-induced fragmentation under matrix isolation conditions: 5-methoxy-1-phenyl-1H-tetrazole (5MPT) [38], 5-ethoxy-1-phenyl-1H-tetrazole (5EPT) [21] and 5-allyloxy-1-phenyl-1H-tetrazole [39]. It was interesting to find that the tetrazolyl ether structure generates a specific fragmentation pattern, which appears to be common for all the derivatives in the class, as depicted in Scheme 4. 

Two main pathways were shown to represent the main cleavage channels for this class (Scheme 4), both involving the dismantlement of the tetrazole core: (i) pathway A represents the most prevalent channel, in this case, involving extrusion of a neutral nitrogen molecule as the primary fragmentation process and leading to a diazirine intermediate, which was a structure first observed and confirmed by our group [28]; (ii) pathway B represents the splitting of the tetrazole ring, leading to the formation of a phenylazide structure and the respective alkylcyanate counterpart, which readily rearranges into the more stable isocyanate form. Phenylazide then undergoes further cleavage through the loss of molecular nitrogen, yielding the intermediate phenylnitrene. Gradual modifications are detected over time on the IR spectrum upon incidence of UV-light, namely the development of a very characteristic band at ca. 1913 cm^−1^, indicating that the singlet phenylnitrene radical readily rearranges into a 1-aza-1,2,4,6-cycloheptatetraene structure through ring expansion [12,28]. This evidence supports our proposal that this more stable structure should also prevail as a final form of the fragment with *m*/*z* 91, detected in EI-MS.

#### 3.2.2. Tetrazolones

**Scheme 5:**
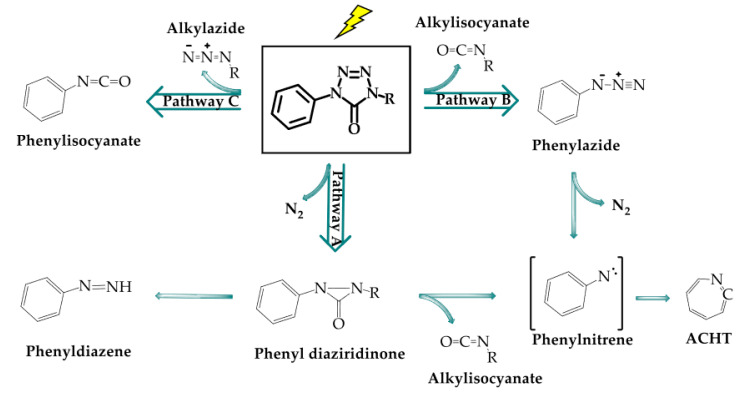
A schematic representation of the main photoreactions induced by UV light (λ > 235 nm) in matrix-isolated tetrazolones.

Photochemical studies previously performed on two tetrazolone derivatives, 1-phenyl-4-allyl-tetrazolone (3a) [40] and 1-phenyl-tetrazolone [12], reveal a more complex photofragmentation scheme than for the tetrazolyl ethers, given the higher lability of the tetrazolone core comparatively to the aromatic tetrazole. Scheme 5 depicts the main fragmentation routes. N_2_ elimination represents the preferred fragmentation channel (pathway A), leading to a diaziridinone species that further cleaves to form a phenyldiazene product by the loss of carbon monoxide in molecules lacking substitution at N4. Additionally, in this pathway, the diaziridinone intermediate was shown to fragment into a phenyl nitrene species, as a secondary cleavage process, by the loss of an isocyanate moiety. Pathway B involves the direct loss of the latter from the tetrazolone core, yielding a phenylazide species that suffers further cleavage to form the phenylnitrene intermediate and ACHT upon the subsequent loss of molecular nitrogen. Pathway C involves tetrazole ring-opening with the formation of a phenylisocyanate and the respective azide. A schematic representation of the aforementioned channels is presented in Scheme 5.

### 3.3. Theoretical Studies

The full assignment of the observed peaks in mass spectrometry is a challenging task, requiring in-depth time-consuming studies of the fragmentation patterns of molecules and related compounds, as small alterations in the nature of the compounds might reflect into a very divergent behaviour. As such, it is often difficult to establish rules for EI-MS interpretation, and frequently, additional tools emerge as useful complementary techniques for structural elucidation.

The use of modern quantum chemistry (QC) has become an art in many fields, benefitting the scientific community with tools that are sometimes irreplicable experimentally. In spectroscopy studies, many tools have now become available to allow for accurate prediction of spectroscopic data, such as NMR and IR, among others, but EI-MS predictions remain very challenging due to the high number of variables that need to be considered as accurate descriptors of molecular behaviours in this environment [41]. Nonetheless, QC provides the ability of calculating with precision certain parameters that can be used as resources to aid the interpretation of EI-MS spectra, namely bond lengths, which indicate the susceptibility of the bonds for cleavage, as well as free Gibbs energies (ΔG) estimates, which assess the plausibility of formation of certain species. This helps to identify the preferred cleavage routes in EI-MS and predict the species formed in each route, especially when more than one plausible species could correspond to equal *m*/*z* values.

Based on DFT(B3LYP)/6-311++G(d,p) bond length calculations performed for 5-methoxy-1-phenyl-1H-tetrazole and 5-ethoxy-1-phenyl-1H-tetrazole, it was possible to ascertain that the bonds between N3 and N4, C5 and N1 and N1 and N2 are the longest ones in the tetrazole core, being, therefore, the most susceptible to cleavage. These conclusions were confirmed through the experimental photolysis studies in which preferential cleavage through pathway A (Scheme 4) was observed for these compounds and further corroborated by the ΔG values calculated for each pathway [21,38]. Calculated energies of 5-phenoxy-1-phenyltetrazole and its photoproducts, at the same level of theory, were in agreement with the experimental results of the photochemically induced cleavage [42], emphasising the importance of such calculations in these type of studies.

As referred previously in this work, many of the fragments obtained through EI-MS on our studied library of compounds are common to more than one fragmentation pathway. It is thus reasonable to assume, based on the conclusions provided by the photochemical studies, that undertaking theoretical calculations for bond length and ΔG predictions in these groups of compounds, as well as others that also cleave in somewhat ambiguous matters, should constitute an important tool for structure and reaction route elucidations in EI-MS studies, allowing for more accurate identification of each of those species and the establishment of rules and patterns for similar molecules or chemotypes.

## 4. Materials and Methods

All of the compounds investigated in this work were synthesized in our laboratory following the approach depicted in Scheme 1, developed previously [31,32,43,44]. All the compounds studied were characterised and described elsewhere [30,43,44,45]. The mass spectra of the studied tetrazoles were obtained on a VG 7070E mass spectrometer by electron ionization (EI) at 70 eV and are presented as supplementary material.

## 5. Conclusions

5-allyloxy-1-aryl-tetrazoles and 4-allyl-1-aryltetrazole-5-ones undergo multiple fragmentations upon photolytic stimuli such as electronic impact or UV irradiation.

Under electron impact, characteristic fragmentations can be ascribed to 5-allyloxy-1-aryl-tetrazoles, following four probable paths that result from: (a) α-cleavage at the C5 ether function; (b) the loss of a neutral nitrogen molecule from the tetrazole core to form a diazirine cycle; (c) ring-opening at the labile N3–N4 and C5–N1 tetrazole bonds and (d) the loss of aryl group at N1. Multiple secondary fragmentation routes then lead to key molecules, such as aryl isocyanate, arylazide, arylnitrene, isocyanic acid and hydrogen azide radical cations, as well as allyl and aryl cations, affording the representative structures associated with the EI fragmentation of this class of tetrazoles.

4-allyl-1-aryltetrazole-5-ones can be obtained by thermally induced sigmatropic rearrangement of 5-allyloxy-1-aryl-tetrazoles. Comparatively to the tetrazolyl ether precursors, tetrazolones are characterised by the loss of aromaticity in the tetrazole core, loss of a labile ether bond at C5 and gain of a stronger C–N bond at N4 of the tetrazole ring. Thus, electron impact fragmentations in this class of compounds are expected to differ from the above class. Primary fragmentation routes for this group include the four pathways referred for the previous and an additional pathway in which the tetrazole core cleaves at the N1–N2 and N4–C5 bonds, leading to direct formation of aryl isocyanate radical cations, which can further suffer the typical secondary cleavage processes. The final species coincide with those ascribed to the previous class.

Regarding the fragmentation patterns in both 5-allyloxy-1-aryl-tetrazoles and 4-allyl-1-aryltetrazole-5-ones, the present work shows that the preferred cleavage channels and resulting species appear to be highly dependent on the nature of the substituents in positions 1 and 5, the electronic effects resulting in route divergence as well as distinct relative intensities of the fragments obtained. Conversely, it is important to emphasize that a rule cannot be established for the EI-MS fragmentation patterns of this class based solely on this feature, as the stability of the resulting fragments–radical cations, cations and radicals–are in many cases the guiding factors towards a preferred route. This is also in agreement with the results obtained for the tetrazolone class, for which key structural modifications occur, while the cleavage patterns do not suffer dramatic variations when compared to the previous class. Additionally, taking into account that both classes of tetrazoles lead to the same final species, and these are simultaneously generated by multiple pathways, complementary techniques arise as beneficial for pathway elucidation as well as accurate identification of the nature and origins of the products, for these molecules and for analogues with similar behaviour. Photochemistry studies on tetrazolyl ethers reveal that the fragmentation of this class upon UV-induced photolysis occurs mainly through the loss of N_2_ and direct ring-opening towards the formation of arylazide and alkyl isocyanate species. In the case of 4-allyl-1-aryltetrazole-5-ones, the same routes can be observed, the difference being the possibility of immediate formation of an arylisocyanate species from the ring-opening step due to a higher fragility of some tetrazolone core bonds compared to those in tetrazolyl ethers. These results are in agreement with the conclusions withdrawn from the EI-MS studies, enabling the assignment of the fragmentation pattern in EI-MS with a higher degree of certainty. Although the photochemical studies do not show exactly coincident behaviours of our studied library of molecules with the ones reported in EI-MS, we attribute the differences to the inherent specificities of each of the applied techniques and consider the use of complementary photochemistry data of relevance in the elucidation of some of the structures also formed upon electron-impact cleavage and confirmation of the structures of the resulting species using the technique that we proposed to scrutinise.

Quantum chemistry studies appear to also provide an efficient tool to validate the results collected through EI-MS spectrometry, as well as UV irradiation, as they allow for ascertainment of the labile bonds prone to fragmentation and overall energies of resulting species, thus contributing to the structural elucidation in such studies.

## Figures and Tables

**Table 1:** Structures, molecular ions [M]^+·^ and significant fragmentary ions registered in the EI-MS spectra of the studied 5-allyloxy-1-aryl-tetrazoles, accompanied by the respective relative abundancies (in parentheses, %).

Tetrazolyl Ether	R^1^	R^2^	Structure	[M]^+·^	Fragmentary Ions
2a	H	H	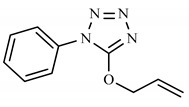	202 (1)	77 (100), 41 (92), 119 (55), 91 (30), 133 (13), 105 (10), 173 (5).
2b	H	CH_3_	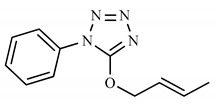	216 (4)	119 (100), 55 (60), 77 (25), 91 (16), 162 (5).
2c	H	C_6_H_5_	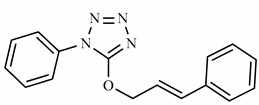	278 (<1)	117 (100), 91 (14), 119 (13), 77 (8), 250 (4).
2d	H	C_7_H_12_	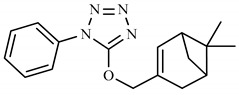	296 (<1)	119 (100), 91 (80), 134 (56), 163 (43), 77 (40), 105 (23), 43 (10).
2e	H	C_6_H_4_NO_2_	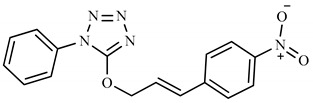	323 (2)	119 (100), 116 (61), 91 (37), 43 (23), 77 (15), 162 (8), 295 (7).
2f	NO_2_	C_6_H_5_	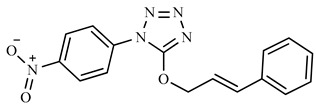	323 (5)	117 (100), 118 (11), 91 (10), 295 (9), 77 (6).
2g	F	C_6_H_5_	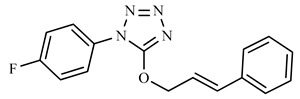	296 (4)	117 (100), 137 (41), 91 (24), 109 (17), 118 (16), 77 (12), 104 (8), 105 (6), 268 (5).
2h	NH_2_	C_6_H_5_	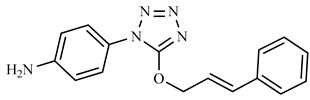	293 (4)	134 (100), 106 (51), 117 (34), 105 (25), 43 (15), 91 (11), 250 (6), 77(5).

**Table 2:** Structures, molecular ions [M]^+·^ and significant fragmentary ions registered in the EI-MS spectra of the studied 4-allyl-1-aryl-tetrazole-5-ones, accompanied by the respective relative abundancies (in parentheses, %).

Tetrazolone	R^1^	R^2^	Structure	[M]^+·^	Fragmentary Ions
3b	H	CH_3_	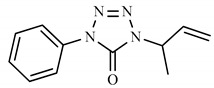	216 (11)	119 (100), 91 (23), 55 (14), 77 (11).
3c	H	C_6_H_5_	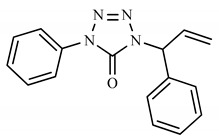	278 (5)	117 (100), 91 (19), 119 (15), 77 (15), 250 (9).
3f	NO_2_	C_6_H_5_	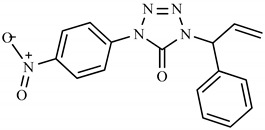	323 (1)	117 (100), 118 (22), 91 (11), 77 (8).
3g	F	C_6_H_5_	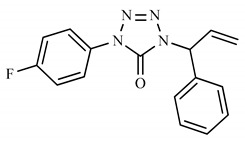	296 (1)	117 (100), 118 (10), 137 (8), 91 (6), 77 (6).
3h	NH_2_	C_6_H_5_	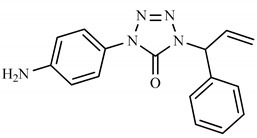	293 (11)	134 (100), 117 (34), 106 (14), 91 (9), 43 (5).

## Data Availability

The data presented in this study are available on request from the corresponding author.

## Supplementary Materials

The following are available online, Figure S1: EI-MS spectrum of 5-(allyloxy)-1-phenyl-1H-tetrazole (2a), Figure S2: EI-MS spectrum of 5-(but-2-en-1-yloxy)-1-phenyl-1H-tetrazole (2b), Figure S3: EI-MS spectrum of 5-(cinnamyloxy)-1-phenyl-1H-tetrazole (2c), Figure S4: EI-MS spectrum of 5-((6,6-dimethylbicyclo[3.1.1]hept-2-en-3-yl)methoxy)-1-phenyl-1H-tetrazole (2d), Figure S5: EI-MS spectrum of 5-((3-(4-nitrophenyl)allyl)oxy)-1-phenyl-1H-tetrazole (2e), Figure S6: EI-MS spectrum of 5-(cinnamyloxy)-1-(4-nitrophenyl)-1H-tetrazole (2f), Figure S7: EI-MS spectrum of 5-(cinnamyloxy)-1-(4-fluorophenyl)-1H-tetrazole (2g), Figure S8: EI-MS spectrum of 4-(5-(cinnamyloxy)-1H-tetrazol-1-yl)aniline (2h), Figure S9: EI-MS spectrum of 1-(but-3-en-2-yl)-4-phenyl-1H-tetrazol-5(4H)-one (3b), Figure S10: EI-MS spectrum of 1-phenyl-4-(1-phenylallyl)-1H-tetrazol-5(4H)-one (3c). Figure S11: EI-MS spectrum of 1-(4-nitrophenyl)-4-(1-phenylallyl)-1H-tetrazol-5(4H)-one (3f), Figure S12: EI-MS spectrum of 1-(4-fluorophenyl)-4-(1-phenylallyl)-1H-tetrazol-5(4H)-one (3g), Figure S13: EI-MS spectrum of 1-(4-aminophenyl)-4-(1-phenylallyl)-1H-tetrazol-5(4H)-one (3h) (amplified 3x).

## Sample Availability

Samples of the compounds are not available from the authors.
