# Supplementary material for: Substituent Effects on EI-MS Fragmentation Patterns of 5-Allyloxy-1-aryl-tetrazoles and 4-Allyl-1-aryl-tetrazole-5-ones; Correlation with UV-Induced Fragmentation Channels
Source: Molecules. 2021 May 29;26(11):3282. doi: 10.3390/molecules26113282 (PMC8199304; doi:10.3390/molecules26113282)
Supplement: Supplementary file 1 [file molecules-26-03282-s001.zip › molecules-1238545-supplementary.pdf]

SUPPORTING INFORMATION

Substituent Effects on EI-MS Fragmentation Patterns of 1-aryl 5-allyloxytetrazoles and 1-aryl 4- allyltetrazole-5-ones; Correlation with UV-Induced Fragmentation Channels

Alina Secrieru [1,2] and RabahOumeddour [1,3] and Maria L. S. Cristiano [1,*]

[1] CCMAR and Department of Chemistry and Pharmacy, FCT, Campus de Gambelas,University of Algarve, 8005-039 Faro, Portugal;
[2] Department of Chemistry, University of Liverpool, Liverpool L69 7ZD, UK
[3] Laboratory of Industrial Analysis and Materials Science, Faculty MISM, University 8 Mai1945, Guelma 24000, Algeria

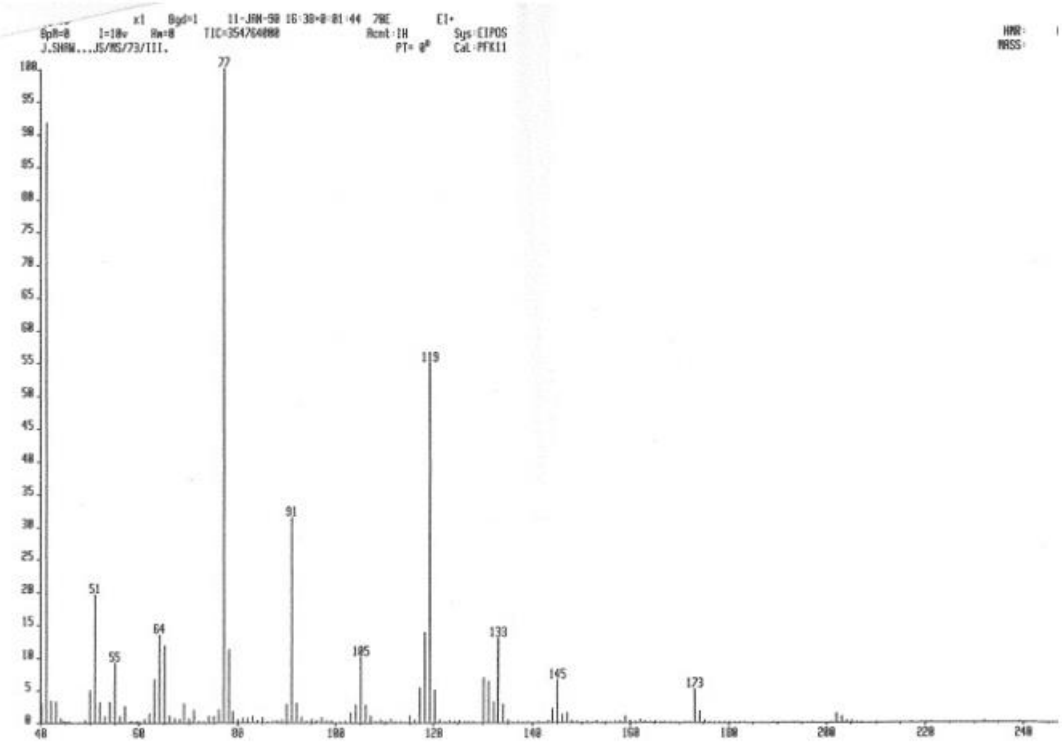

Figure S1. - EI-MS spectrum of 5-(allyloxy)-1-phenyl-1H-tetrazole (2a).

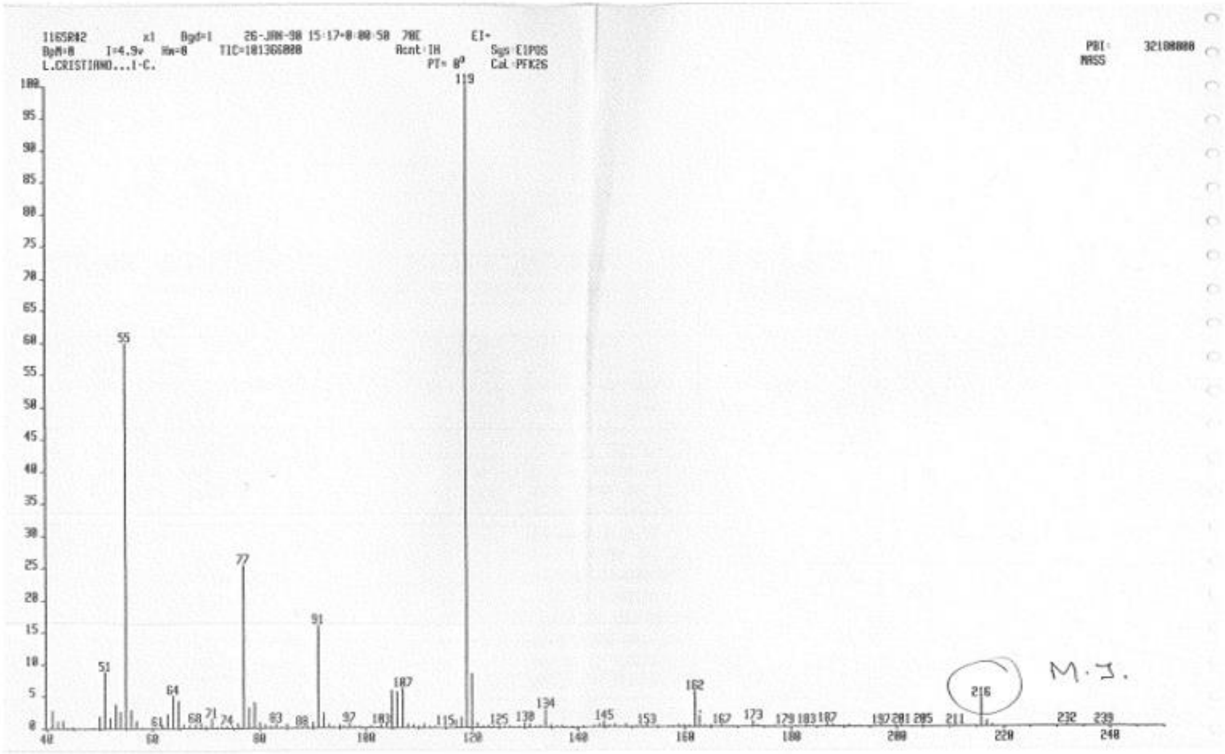

Figure S2. - EI-MS spectrum of 5-(but-2-en-1-yloxy)-1-phenyl-1H-tetrazole (2b).

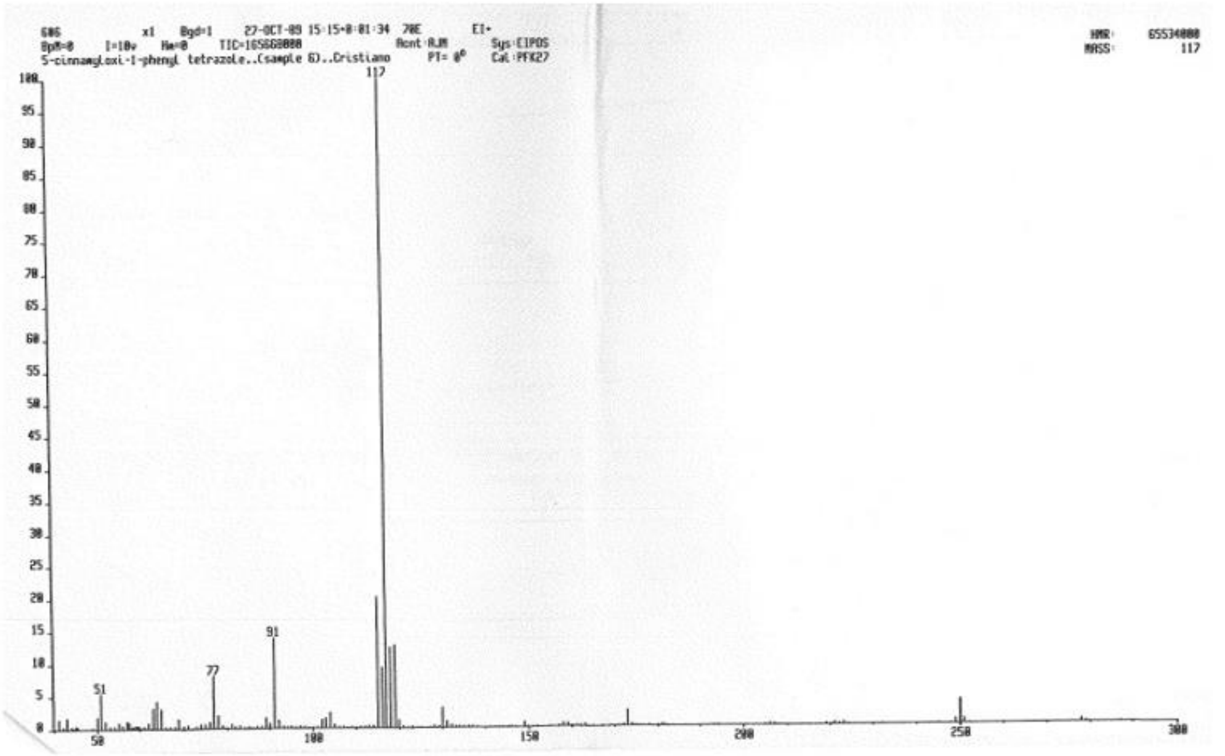

Figure S3. - EI-MS spectrum of 5-(cinnamyloxy)-1-phenyl-1H-tetrazole (2c).

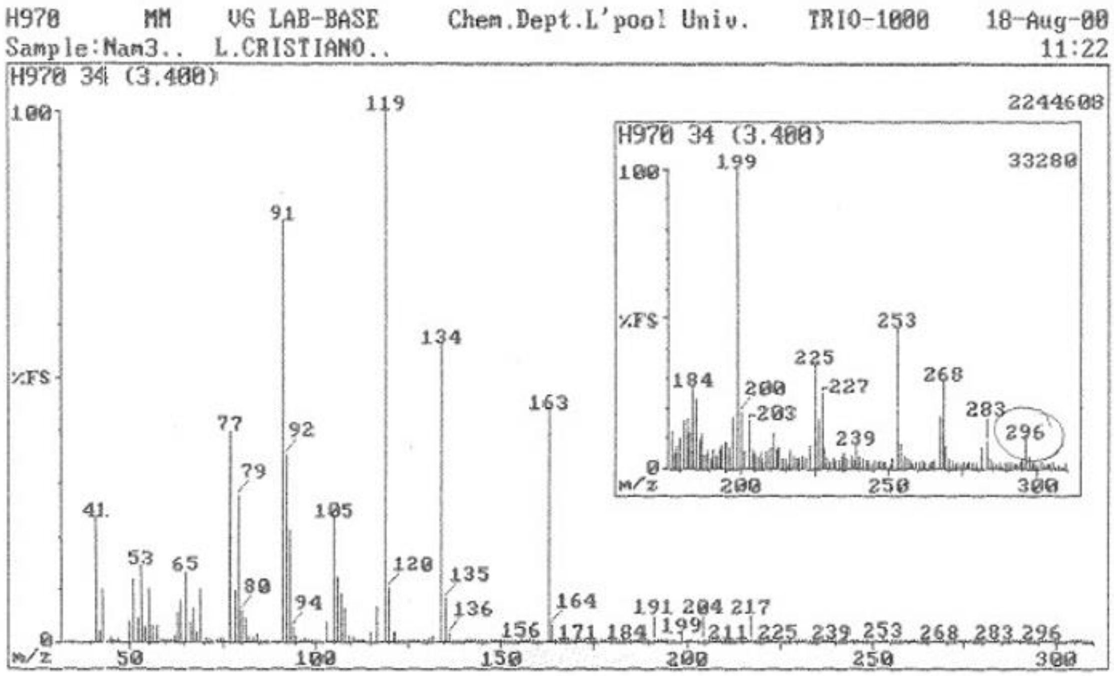

Figure S4. - EI-MS spectrum of 5-((6,6-dimethylbicyclo[3.1.1]hept-2-en-3-yl)methoxy)-1-phenyl-1H-tetrazole (2d).

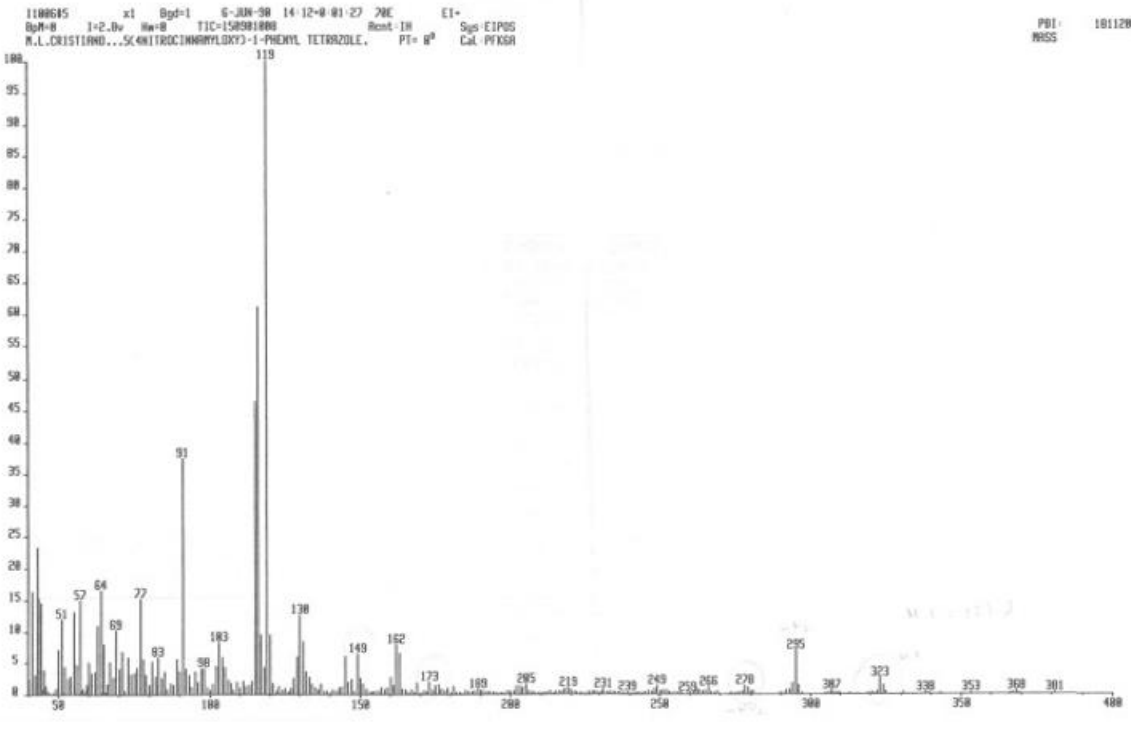

Figure S5. - EI-MS spectrum of 5-((3-(4-nitrophenyl)allyl)oxy)-1-phenyl-1H-tetrazole (2e).

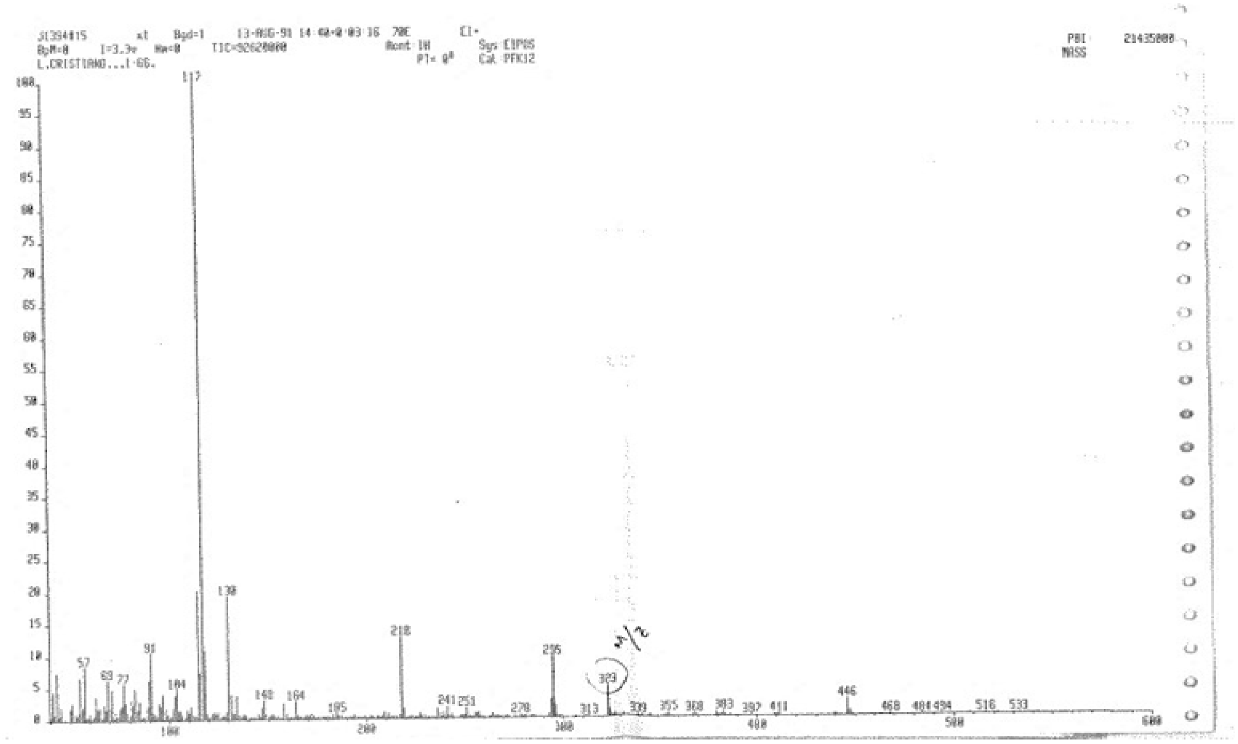

Figure S6. - EI-MS spectrum of 5-(cinnamyloxy)-1-(4-nitrophenyl)-1H-tetrazole (2f).

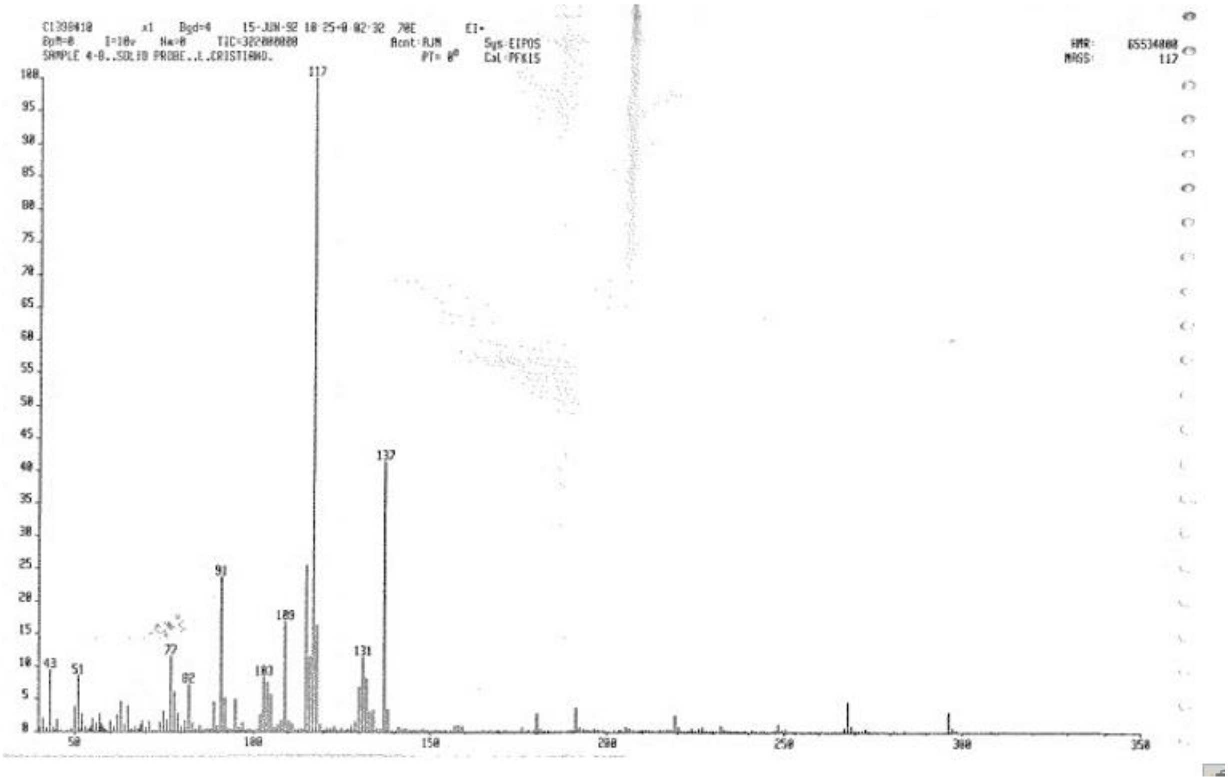

Figure S7. - EI-MS spectrum of 5-(cinnamyloxy)-1-(4-fluorophenyl)-1H-tetrazole (2g).

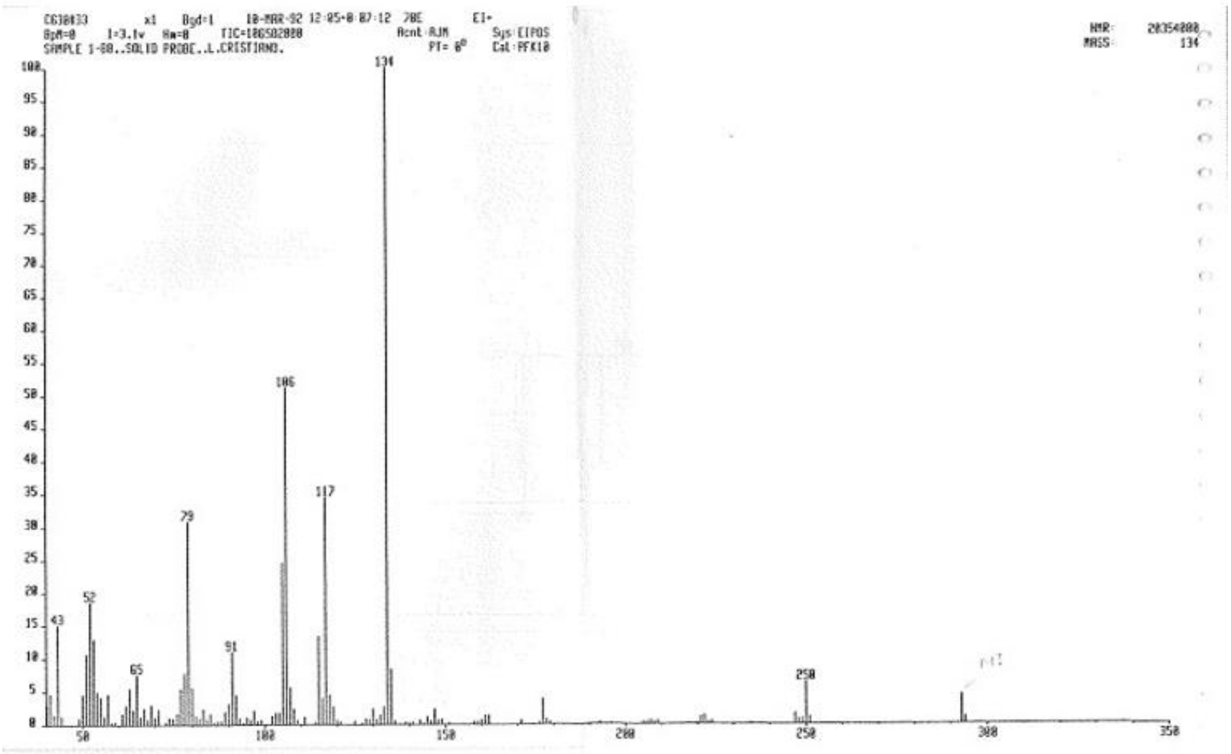

Figure S8. - EI-MS spectrum of 4-(5-(cinnamyloxy)-1H-tetrazol-1-yl)aniline (2h).

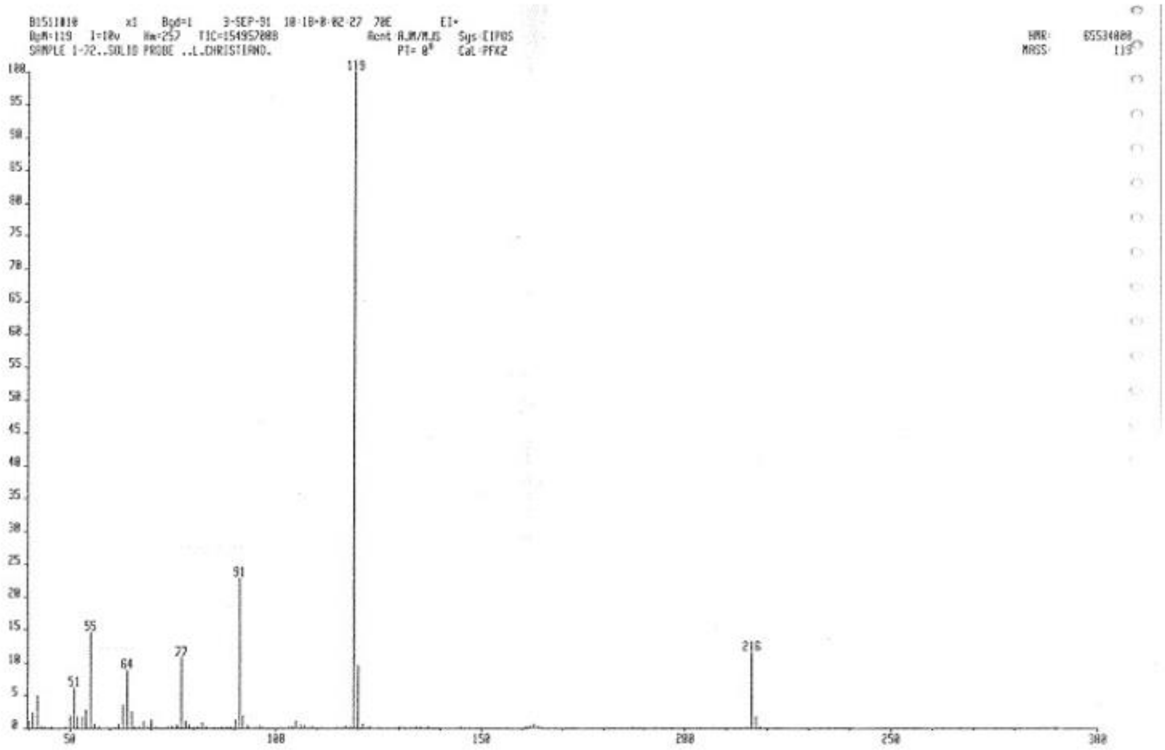

Figure S9. - EI-MS spectrum of 1-(but-3-en-2-yl)-4-phenyl-1H-tetrazol-5(4H)-one (3b).

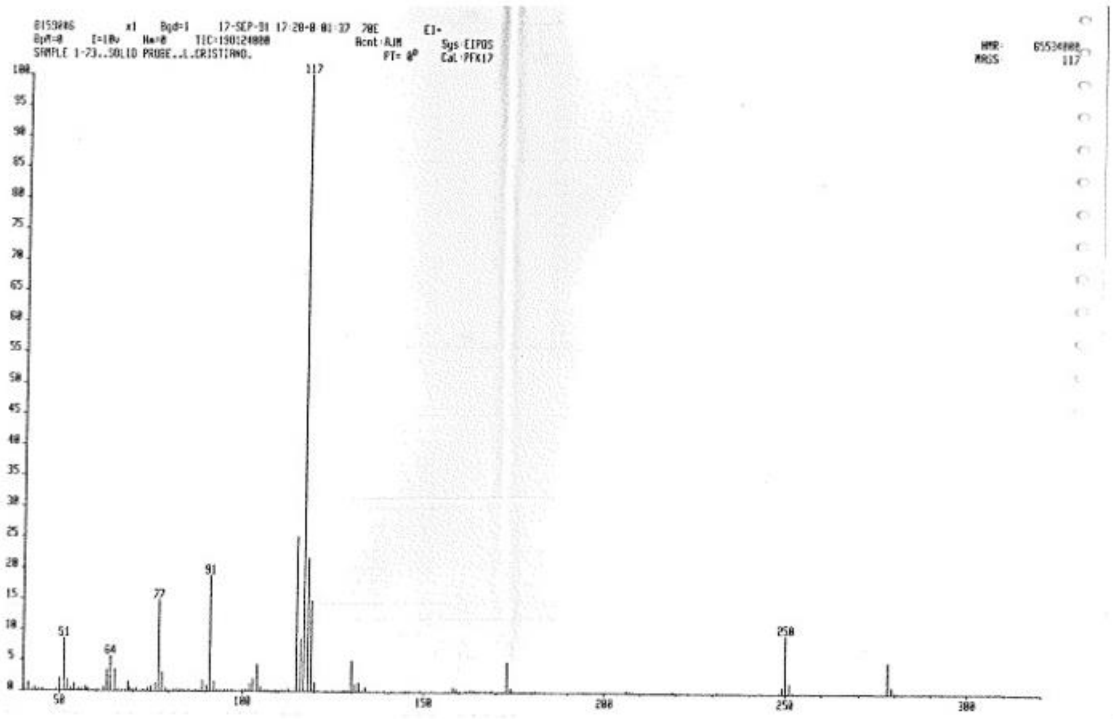

Figure S10. - EI-MS spectrum of 1-phenyl-4-(1-phenylallyl)-1H-tetrazol-5(4H)-one (3c).

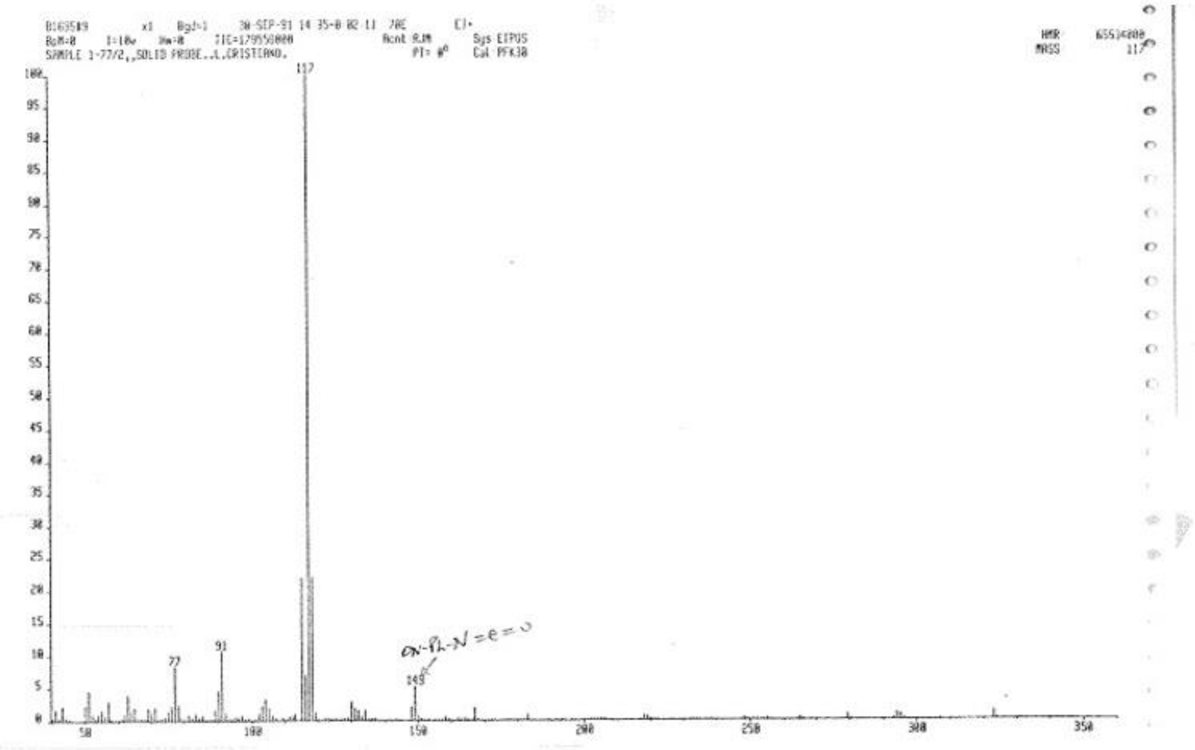

Figure S11. - EI-MS spectrum of 1-(4-nitrophenyl)-4-(1-phenylallyl)-1H-tetrazol-5(4H)-one (3f).

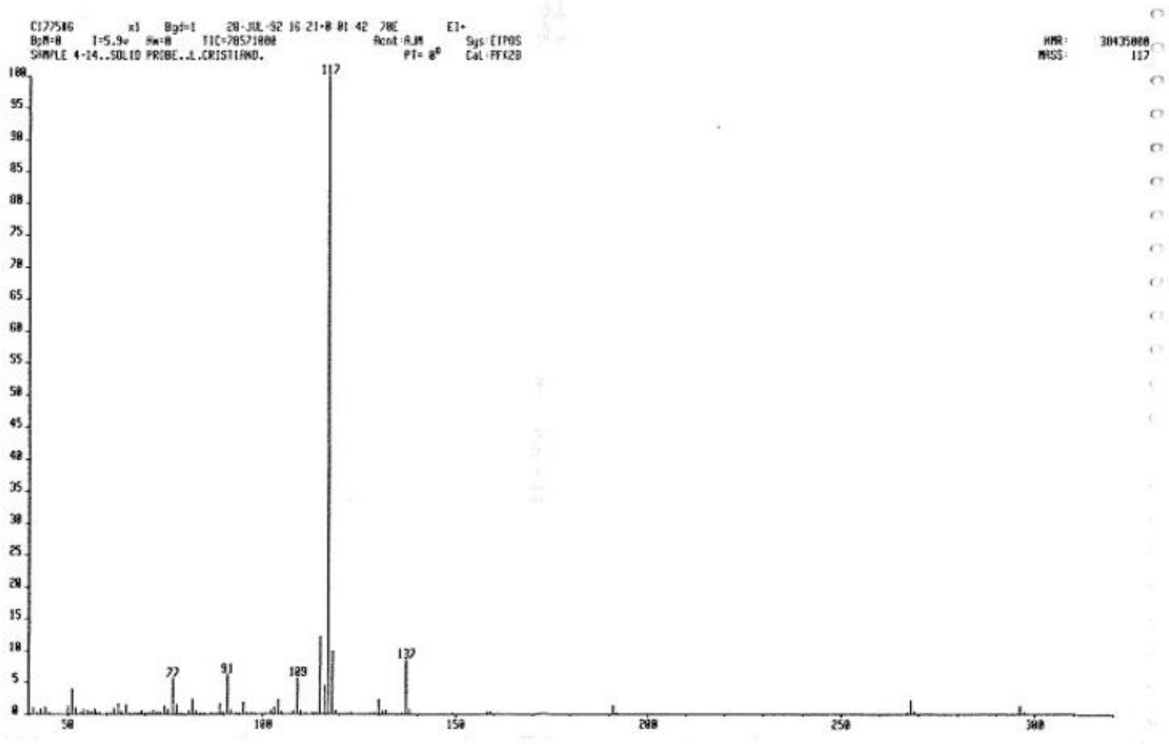

Figure S12. - EI-MS spectrum of 1-(4-fluorophenyl)-4-(1-phenylallyl)-1H-tetrazol-5(4H)-one (3g).

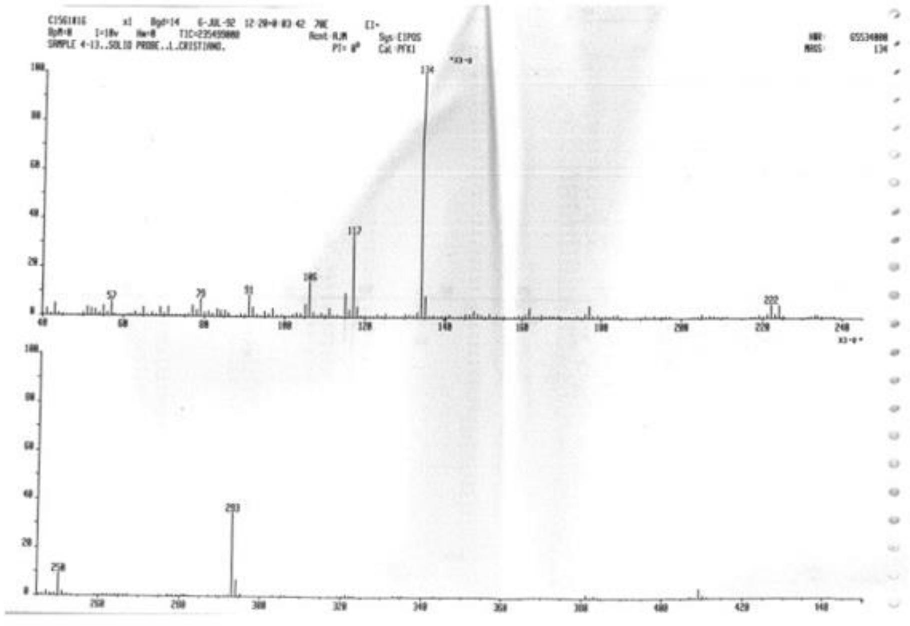

Figure S13. - EI-MS spectrum of 1-(4-aminophenyl)-4-(1-phenylallyl)-1H-tetrazol-5(4H)-one (3h)(amplified 3x).
